# The Impact of a New Internal Medicine Residency Program on Patient Satisfaction Scores for Teaching Hospitalist Faculty Compared to Non-teaching Hospitalist

**DOI:** 10.7759/cureus.20795

**Published:** 2021-12-29

**Authors:** Janeane Walker, John E Delzell

**Affiliations:** 1 Graduate Medical Education, Northeast Georgia Medical Center, Gainesville, USA

**Keywords:** hospitalist medicine, resident-patient communication, provider-patient communication, patient experience, patient satisfaction, hospital consumer assessment

## Abstract

Introduction: The Hospital Consumer Assessment of Healthcare Providers and Systems (HCAHPS) is a national survey sent to patients to measure their inpatient experience. Graduate medical education programs may affect a sponsoring institution in various ways, but there has been little research into the effect of teaching hospitalist faculty on HCAHPS scores in a community-based hospital. The aim of the current study is to evaluate if the introduction of internal medicine resident physicians would affect the HCAHPS scores of patients admitted by hospitalist faculty physicians.

Methods: This was a retrospective analysis of anonymous patient satisfaction survey data for internal medicine hospitalist teams from January 2019 to December 2019. Data were retrieved from the Press Ganey database. We compared two groups: teaching hospitalists (N = 12) and non-teaching hospitalists (N = 34). Data were divided into two time periods: January to June (pre-residents) and July to December (post-residents).

Results: From January to June (pre-residents), 646 HCAHPS surveys were returned. For the post-resident cohort (July to December), a total of 487 surveys were returned. The “Recommend” domain, showed a significant improvement in the mean pre-resident to post-resident (57% to 69%; p = 0.0351).

Conclusion: There was a significant increase in the mean rating of the “Recommend” hospital domain for the teaching hospitalists when compared to the non-teaching after the addition of a new internal medicine residency program.

## Introduction

There is a burgeoning number of new graduate medical education (GME) programs that have been accredited over this last decade [1]. This provides an opportunity to study how the introduction of residents into GME naïve settings can influence the Hospital Consumer Assessment of Healthcare Providers and Systems (HCAHPS) scores of physician teaching hospitalists versus non-teaching hospitalists. The HCAHPS survey is used to measure communication and quality of patient care [2]. This validated survey is required by the Centers for Medicare & Medicaid Services (CMS) for hospitals receiving funding [3]. HCAHPS data are often used for hospital benchmarking of public perception surrounding the delivery of care [4-5]. The survey is sent to randomly selected, discharged patients. It is comprised of 29 questions about the patients’ overall hospital experience [6]. The three domains on the HCAHPS survey about provider-patient communication center on courtesy and respect, the careful listening of doctors, and explanation of care [3]. For the overall rating of the hospital, there is a global score from 0 to 10 and willingness to recommend the hospital. This study looked at two specific composite domains: provider-patient communication and the overall ranking as well as the additional physician-related question measuring “explanations provided by the hospitalist.”

Previous studies have evaluated interventions to improve HCAHPS scores. In 2017, Seiler et al. found that a simulation-based communication curriculum introduced to medical staff did not significantly improve physician-related HCAHPS scores [7]. In contrast, Allenbaugh et al. found that physician-related HCAHPS scores improved when resident physicians received education on communication skills [8]. While Banka et al. found that resident physicians who were provided with individualized patient feedback had significant improvement in their patient satisfaction scores [9]. A 2017 systematic review by Davidson et al. recommended more generalizable interventions to improve HCAHPS and patient satisfaction scores [10].

Patient experience and the quality of care provided by resident physicians are important for faculty in GME training programs. Faculty physicians are responsible for the education of residents and resident success as measured by milestone outcomes. Milestones for physician education are divided into six core competencies surrounding patient care, medical knowledge, professionalism, interpersonal and communication skills, practice-based learning, and systems-based practice across all specialty programs [11]. An assessment comparing the HCAHPS communication questions to the Accreditation Council for Graduate Medical Education (ACGME) internal medicine milestones found that there were no milestone questions related to understanding and listening to the patient or being understood by the patient [12]. Additional studies have shown that hospitalists overall have higher patient satisfaction scores and improvements in length of stay compared to non-hospitalists [13]. The purpose of this study was to evaluate if the introduction of internal medicine resident physicians would affect the HCAHPS scores of our physicians serving as faculty (teaching hospitalists) in the internal medicine residency program as compared to non-teaching hospitalists.

## Materials and methods

The Brenau University Institutional Review Board (IRB) determined that the study meets exemption criteria. The approval number from the IRB is 1573811-1. This article does not contain any studies with human or animal subjects. There are no human subjects in this article and informed consent is not applicable.

A retrospective time-sequential cohort study was conducted. An analysis of anonymous patient satisfaction survey data from January 2019 to December 2019 was completed. HCAHPS data were retrieved from the database of our vendor Press Ganey. We compared two groups: teaching hospitalists (N = 12) and non-teaching hospitalists (N = 34) (see Table 1). There were no changes in the number of hospitalists in either cohort group over the study period. All teaching hospitalists had a resident on their inpatient team while the comparison group was without physician residents. All patient survey data admitted by internal medicine hospitalists to Northeast Georgia Medical Center (NGMC) were included.

**Table 1:** Demographics of the attending hospitalist physicians. ABIM, American Board of Internal Medicine.

	Teaching physicians	Non-teaching physicians
Total physicians	12	34
Male	9 (75%)	22 (65%)
Female	3 (25%)	12 (35%)
Age (average)	38.9	41.0
Years in practice (average)	6.2	8.85
Board certification (ABIM)	100%	100%

Information surrounding the inaugural internal medicine program and interest to serve as GME internal medicine faculty was presented to all hospitalists by the internal medicine program director. The program director discussed the roles and responsibilities that would come as serving as faculty. Interested faculty contacted the program director for internal medicine in which formal interviews were conducted. After the selection of GME faculty, the program director held a series of faculty development sessions. The internal medicine faculty selected did not have prior GME teaching experience. As part of the ACGME program requirements for GME in internal medicine, it is the role of the program director to ensure that each chosen faculty person has met the criteria as set forth by the ACGME to serve as faculty [14]. The program director is also responsible for continued training of faculty and conducts faculty evaluations annually. The GME faculty did not receive additional compensation. Their role as faculty was integrated into their current existing contract for the hospital.

Northeast Georgia Health System (NGHS) is a not-for-profit community health system with a service area of more than 1 million people across 18 counties through four hospitals and a variety of outpatient locations. NGMC Gainesville is a tertiary care center with 560 beds and more than 800 medical staff members representing over 50 specialties. NGMC is a new ACGME sponsoring institution with an inaugural class of 20 internal medicine resident physicians who began July 1, 2019. Prior to July 1, 2019, there were no resident physicians at NGMC Gainesville. This provided a unique opportunity to measure the change in patient satisfaction as measured by HCAHPS scores before and after resident physicians arrived for our teaching hospitalist serving as GME faculty.

Prior to the introduction of residents, employed internal medicine hospitalist physicians would care for an average of 18 patients per day (range 15 to 21 patients). All patients were admitted by either a nocturnist or admitting internist. There were 8-12 non-teaching hospitalist rounding teams, depending on hospital census. On July 1, 2019, a new inpatient teaching service structure was created for the internal medicine residents, consisting of four inpatient teams. Each team consisted of a teaching hospitalist with two interns. The teaching hospitalist teams average 15 patients (range 12 to 18 patients). All of the teaching hospitalists were a part of the employed hospitalist group. The teaching and non-teaching hospitalists were all admitting patients for the entire period studied.

Survey data were divided into two time periods: January to June (pre-residents) and July to December (post-residents). All hospitalist physicians studied were admitting patients across the entire time frame. Global hospital rating and physician-related questions were examined (see Table 2). For the rating question, we considered the percentage that was rated in the top box score percentages from 9 to 10. For all other questions, we compared the percentage that answered “always.” Statistical data were analyzed with StatCrunch software (Pearson Education, London, UK). Statistical significance was defined as p < 0.05.

**Table 2:** HCAHPS survey questions. HCAHPS, Hospital Consumer Assessment of Healthcare Providers and Systems.

Question	Scale
Global	
“What number would you use to rate this hospital during your stay?”	0 to 10
“Would you recommend this hospital to your friends and family?”	Definitely no, probably no, probably yes, definitely yes
Physician	
“During this hospital stay, how often did doctors treat you with courtesy and respect?”	Never, sometimes, usually, always
“During this hospital stay, how often did doctors listen carefully to you?”	Never, sometimes, usually, always
“During this hospital stay, how often did doctors explain things in a way you could understand?”	Never, sometimes, usually, always
Additional hospitalist specific questions	
“How were the explanations provided by the hospitalist?”	Very poor, poor, fair, good, very good
“The degree to which the hospitalist explained their role, filling in for your primary care physician?”	Very poor, poor, fair, good, very good

## Results

From January to June (pre-resident), 646 surveys were completed and returned. This included 174 surveys for teaching hospitalists and 472 surveys for non-teaching hospitalists. For the post-resident cohort (July to December), there were a total of 487 surveys returned. There were 145 surveys for teaching hospitalists and 342 surveys for non-teaching hospitalists. In the “Recommend NGHS” domain, there was a significant improvement in the pre-resident to post-resident mean (57%-69%; p = 0.0351). The “Hospitalist explained role” domain was significantly worse in the non-teaching group (54%-47%; p = 0.0257). The overall rating of the hospital by patients seen by the teaching hospitalists improved after the addition of resident physicians (60%-66%, p = 0.06). A composite of all eight questions was not significantly different before and after residents (p = 0.4144). The teaching hospitalist group had non-significant increases in pre-residents to post-residents on three other questions including “Doctors listen” (70%-77%), “Doctors explain,” and “Explanation provided by the hospitalist.” The non-teaching hospitalist group had some improvement in those areas but to a lesser extent. There was a significant increase in the mean rating by patients in the “Recommend NGHS” domain for the teaching hospitalists when compared to the non-teaching (Table 3).

**Table 3:** HCAHPS report: January 2019 to December 2019. Software StatCrunch is used for the paired T hypothesis test output. μ1 represents the mean rating for “attending with residents” from January 2019 to December 2019. μ2 represents the mean rating for “attending without residents” from January 2019 to December 2019. HCAHPS, Hospital Consumer Assessment of Healthcare Providers and Systems; NGHS, Northeast Georgia Health System.

Rating question	Ho	Ha	Paired T hypothesis test output						Statistical conclusion
			Difference	Mean	Standard error	DF	T-stat	P-value	
Rate 9-10	μ_1 _= μ_2_	μ_1 _< μ_2_	μ_1_-μ_2_	0.56666667	3.0521809	11	0.1856596	0.572	No statistical significance
Recommend NGHS	μ_1 _= μ_2_	μ_1 _< μ_2_	μ_1_-μ_2_	−0.15833333	3.514051	11	−0.04505721	0.9649	No statistical significance
	μ_1 _= μ_2_	μ_1 _> μ_2_	μ_1_-μ_2_	−0.15833333	3.514051	11	−0.04505721	0.0351	Statistical significant
Communication with doctors	μ_1 _= μ_2_	μ_1 _< μ_2_	μ_1_-μ_2_	0.70833333	2.5605237	11	0.27663612	0.6064	No statistical significance
Doctors courteous/respectful	μ_1 _= μ_2_	μ_1 _< μ_2_	μ_1_-μ_2_	1.6	2.8070528	11	0.56999284	0.7099	No statistical significance
Doctors listen	μ_1 _= μ_2_	μ_1 _< μ_2_	μ_1_-μ_2_	2.3416667	3.0558057	11	0.76630091	0.7702	No statistical significance
Doctors explain	μ_1 _= μ_2_	μ_1 _< μ_2_	μ_1_-μ_2_	−1.7666667	2.9496105	11	−0.59894914	0.2807	No statistical significance
Hospitalist explained role	μ_1 _= μ_2_	μ_1 _< μ_2_	μ_1_-μ_2_	−5.725	2.6209565	11	−2.1843171	0.0257	Statistical significant
Explanation provided by hospitalist	μ_1 _= μ_2_	μ_1 _< μ_2_	μ_1_-μ_2_	0.575	3.7590724	11	0.15296327	0.5594	No statistical significance
All eight questions	μ _1_= μ_2_	μ_1 _< μ_2_	μ_1_-μ_2_	−0.23229167	1.070667	95	−0.21695977	0.4144	No statistical significance

## Discussion

This study aimed to demonstrate changes in the patient experience scores between teaching hospitalists compared to non-teaching hospitalists before and after the introduction of a new internal medicine residency program. This study showed increases in HCAHPS scores of our teaching faculty who worked with resident physicians on the inpatient hospital service. We did not show improvements in all the physician-related HCAHPS questions for teaching hospitalists. The p-value of “Recommend NGHS” is statistically significant while comparing “attending with residents” being greater than “attending without residents” during the time period January 2019 to December 2019, and the p-value of “Hospitalist explained role” is statistically significant while comparing “attending with residents” being less than “attending without residents” during the time period January 2019 to December 2019. This means that the mean rating of “Recommend NGHS” for “attending with residents” is better than the one for “attending without residents” during that time period, and the mean rating of “Hospitalist explained role” for “attending with residents” is worse than the one for “attending without residents” during that time period.

Supporting our work, Iannuzzi et al. compared resident team’s patient satisfaction data with advanced practice clinical teams, showing higher patient satisfaction scores as well as a decrease in the patient’s length of stay for the physician residents’ teams [15]. These data suggest that residents can impact more than just the perception of care received but not other quality hospital indicators. Lappe et al. demonstrated that hospitalist team structure can impact patient satisfaction [16]. Their results, in contrast to our study, showed that patients had more satisfaction with physicians on solo hospitalist teams as compared to hospitalist teams with residents and advanced practice providers. For our study, the addition of resident physicians changed the structure of our inpatient hospitalist teams. This supports the need to further investigate the overall influence that residents bring to the patient care team longitudinally.

In contrast with our study, previous work by Wray et al. found that non-teaching hospitalists had better patient satisfaction scores than the teaching hospitalist [17]. In comparison, our validated survey instrument differed from the validated survey instrument utilized in Wray et al.'s study. Although there are differences in the results, this further suggests that many factors can play into the overall patient satisfaction scores for hospitals.

There are several limitations to this study. First, this study was conducted at a single community-based medical center. It is unclear if the results can be generalized to other institutions and specialties beyond internal medicine. Second, this study used a historical control group (the six months prior to resident physician arrival). The hospitalist physician faculty members (teaching and non-teaching) remained the same throughout the entire study period. There was no crossover between the physicians who were teaching and those who were not, and they all stayed in their group for the study period. Third, the selected faculty did not have previous GME teaching experience nor did they receive additional compensation. Although the program director held a faculty development session, the confounding variable of not having taught in a GME prior may influence the HCAHPS data. The final limitation is related to the specific way that the HCAHPS survey is administered. The discharging physician is listed as the provider on the HCAHPS survey. It is important to note that the discharging physician may not have been the physician caring for the patient during their entire hospital stay. The hospital system has used HCAHPS for many years and the methodology for collecting surveys has been unchanged.

## Conclusions

In conclusion, our intervention utilized a natural experiment created by the addition of internal medicine residents into our hospital system. This may inform organizational leaders of the potential value that teaching faculty have on the patient perception of the patient experience. This study offers the opportunity to take a deeper look at the communication dynamics between the provider-patient relationship, the attending-resident interaction, and the resident-patient communication. There is a need for further study on the impact of resident physicians’ specific data on patient satisfaction including ways to teach and improve physician communication skills.

## References

[1] Hayek S, Lane S, Fluck M, Hunsinger M, Blansfield J, Shabahang M (2018). Ten year projections for US residency positions: will there be enough positions to accommodate the growing number of U.S. medical school graduates?. J Surg Educ.

[2] Tefera L, Lehrman WG, Conway P (2016). Measurement of the patient experience: clarifying facts, myths, and approaches. JAMA.

[3] (2021). Hospital CAHPS (HCAHPS). https://www.cms.gov/Research-Statistics-Data-and-Systems/Research/CAHPS/HCAHPS1.

[4] (2021). HCAHPS: patients' perspectives of care survey. https://www.cms.gov/Medicare/Quality-Initiatives-Patient-Assessment-Instruments/HospitalQualityInits/HospitalHCAHPS.

[5] Chatterjee P, Tsai TC, Jha AK (2015). Delivering value by focusing on patient experience. Am J Manag Care.

[6] (2021). HCAHPS fact sheet. https://www.hcahpsonline.org/en/facts/.

[7] Seiler A, Knee A, Shaaban R (2017). Physician communication coaching effects on patient experience. PLoS One.

[8] Allenbaugh J, Corbelli J, Rack L, Rubio D, Spagnoletti C (2019). A brief communication curriculum improves resident and nurse communication skills and patient satisfaction. J Gen Intern Med.

[9] Banka G, Edgington S, Kyulo N (2015). Improving patient satisfaction through physician education, feedback, and incentives. J Hosp Med.

[10] Davidson KW, Shaffer J, Ye S (2017). Interventions to improve hospital patient satisfaction with healthcare providers and systems: a systematic review. BMJ Qual Saf.

[11] (2021). ACGME. Common program requirements (residency). https://www.acgme.org/Portals/0/PFAssets/ProgramRequirements/CPRResidency2020.pdf.

[12] Sanford B, Whitehouse S, Kokas M (2017). Do ACGME physician-patient communication milestones align with HCAHPS patient satisfaction measures for doctor communication?. Am J Med Qual.

[13] Salim SA, Elmaraezy A, Pamarthy A, Thongprayoon C, Cheungpasitporn W, Palabindala V (2019). Impact of hospitalists on the efficiency of inpatient care and patient satisfaction: a systematic review and meta-analysis. J Community Hosp Intern Med Perspect.

[14] (2021). ACGME program requirements for graduate medical education in internal medicine. https://www.acgme.org/globalassets/pfassets/programrequirements/140_internalmedicine_2020.pdf.

[15] Iannuzzi MC, Iannuzzi JC, Holtsbery A, Wright SM, Knohl SJ (2015). Comparing hospitalist-resident to hospitalist-midlevel practitioner team performance on length of stay and direct patient care cost. J Grad Med Educ.

[16] Lappé KL, Raaum SE, Ciarkowski CE, Reddy SP, Johnson SA (2020). Impact of hospitalist team structure on patient-reported satisfaction with physician performance. J Gen Intern Med.

[17] Wray CM, Flores A, Padula WV, Prochaska MT, Meltzer DO, Arora VM (2016). Measuring patient experiences on hospitalist and teaching services: patient responses to a 30-day postdischarge questionnaire. J Hosp Med.

